# Investigation on absorption cross-section of photosynthetic pigment molecules based on a mechanistic model of the photosynthetic electron flow-light response in C_3_, C_4_ species and cyanobacteria grown under various conditions

**DOI:** 10.3389/fpls.2023.1234462

**Published:** 2023-08-29

**Authors:** Zi-Piao Ye, Alexandrina Stirbet, Ting An, Piotr Robakowski, Hua-Jing Kang, Xiao-Long Yang, Fu-Biao Wang

**Affiliations:** ^1^ The Institute of Biophysics in College of Mathematics and Physics, Jinggangshan University, Ji’an, Jiangxi, China; ^2^ Retired, Newport News, VA, United States; ^3^ School of Biological Sciences and Engineering, Jiangxi Agriculture University, Nanchang, China; ^4^ Faculty of Forestry and Wood Technology, Poznan University of Life Sciences, Poznan, Poland; ^5^ Southern Zhejiang Key Laboratory of Crop Breeding, Wenzhou Academy of Agricultural Sciences, Wenzhou, Zhejiang, China; ^6^ School of Life Sciences, Nantong University, Nantong, Jiangsu, China

**Keywords:** absorption cross-section, effective absorption cross-section, environmental factors, photosynthetic pigment molecules, photosynthesis

## Abstract

Investigation on intrinsic properties of photosynthetic pigment molecules participating in solar energy absorption and excitation, especially their eigen-absorption cross-section (*σ*_ik_) and effective absorption cross-section (*σ* ^′^_ik_), is important to understand photosynthesis. Here, we present the development and application of a new method to determine these parameters, based on a mechanistic model of the photosynthetic electron flow-light response. The analysis with our method of a series of previously collected chlorophyll *a* fluorescence data shows that the absorption cross-section of photosynthetic pigment molecules has different values of approximately 10^−21^ m^2^, for several photosynthetic organisms grown under various conditions: (1) the conifer *Abies alba* Mill., grown under high light or low light; (2) *Taxus baccata* L., grown under fertilization or non-fertilization conditions; (3) *Glycine max* L. (Merr.), grown under a CO_2_ concentration of 400 or 600 μmol CO_2_ mol^−1^ in a leaf chamber under shaded conditions; (4) *Zea mays* L., at temperatures of 30°C or 35°C in a leaf chamber; (5) *Osmanthus fragrans* Loureiro, with shaded-leaf or sun-leaf; and (6) the cyanobacterium *Microcystis aeruginosa* FACHB905, grown under two different nitrogen supplies. Our results show that *σ*_ik_ has the same order of magnitude (approximately 10^−21^ m^2^), and *σ* ^′^_ik_ for these species decreases with increasing light intensity, demonstrating the operation of a key regulatory mechanism to reduce solar absorption and avoid high light damage. Moreover, compared with other approaches, both *σ*_ik_ and *σ* ^′^_ik_ can be more easily estimated by our method, even under various growth conditions (e.g., different light environment; different CO_2_, NO_2_, O_2_, and O_3_ concentrations; air temperatures; or water stress), regardless of the type of the sample (e.g., dilute or concentrated cell suspensions or leaves). Our results also show that CO_2_ concentration and temperature have little effect on *σ*_ik_ values for *G. max* and *Z. mays*. Consequently, our approach provides a powerful tool to investigate light energy absorption of photosynthetic pigment molecules and gives us new information on how plants and cyanobacteria modify their light-harvesting properties under different stress conditions.

## Introduction

In the primary reaction process of photosynthesis, light is absorbed by the light-harvesting pigments including chlorophyll *a*. At room temperature, chlorophyll *a* molecules in their excited state return to the ground state via fluorescence emission and thermal dissipation processes, but most of the excitation energy is transferred to the photosystem II (PSII) and the photosystem I (PSI) to drive photochemistry and charge separation (Mar and Govindjee, 1972; Govindjee, 1990; Bixon et al., 1991; Govindjee, 2004; Wang et al., 2007; Baker, 2008; Grayson et al., 2017; Stirbet et al., 2020). The physical processes of light absorption and other photochemical processes, involving photosynthetic pigment molecules (chlorophyll and carotenoid molecules in plants and green algae), have been extensively studied both theoretically and experimentally due to their importance in photosynthesis (Ley and Mauzerall, 1982; Stirbet et al., 1998; Govindjee, 2004; Walters, 2005; De Boni et al., 2007; Suggett et al., 2007; Baker, 2008; Ye et al., 2013a; Ye et al., 2013b; Klughammer and Schreiber, 2015; Mishra et al., 2019; Stirbet et al., 2019; Hu et al., 2021; Celebi-Ergin et al., 2022; He et al., 2022).

Previous studies have mainly focused on investigating the relationship between the chlorophyll content and the plant’s light absorption and excitation capacity, without considering the impact of parameters such as the intrinsic and effective absorption cross-sections of photosynthetic pigment molecules on this process (Baker, 2008; Ahammed et al., 2018; Robakowski et al., 2018). However, the intrinsic properties of photosynthetic pigment molecules in light absorption and excitation are essential to understand the light-harvesting mechanism in photosynthetic organisms. Between different parameters studied, the following are essential to understand this process: (1) the effective absorption cross-sections of photosystem II (PSII) (*σ*_PSII_) (Suggett et al., 2007; Klughammer and Schreiber, 2015; see **Table 1** for the list of abbreviations); (2) the absorption cross-section for oxygen production ( ${\text{σ}}_{{\text{O}}_{2}}$ ) (Ley and Mauzerall, 1982); (3) the absorption cross-section of chlorophyll molecules (*σ*_chl_) (Ley and Mauzerall, 1982; Suggett et al., 2007); (4) the eigen-absorption cross-section (*σ*_ik_, from the ground state to the first excited state) (Ye et al., 2013a; Ye et al., 2013b; Zuo et al., 2019; Hashemi and Leppert, 2021; Hu et al., 2021; He et al., 2022); (5) the effective absorption cross-section of photosynthetic pigment molecules (*σ* ^′^_ik_) (Ye et al., 2013a; Ye et al., 2013b; Hu et al., 2021; He et al., 2022); and (6) the optical cross-section (*a*_L_*(λ), where λ is the wavelength) (Celebi-Ergin et al., 2022). These absorption cross-sections measured in cyanobacteria, algae, and plants vary among taxa and/or with various growth conditions, as shown in the studies mentioned above.

**Table 1:** Definitions of the abbreviations.

Abbreviation	Definition	Units
*ETR*	Electron transport rate	μmol electrons m^−2^ s^−1^
*ETR–I*	Light–response curve of electron transport	
*ETR*_max_	Maximum electron transport rate	μmol electrons m^−2^ s^-1^
*g*_i_	Degeneration of energy level of photosynthetic pigment molecules in the ground state *i*	Dimensionless
*g*_k_	Degeneration of energy level of photosynthetic pigment molecules in the excited state *k*	Dimensionless
*I*	Light intensity	μmol photons m^−2^ s^−1^
*N*_0_	Total photosynthetic pigment molecules of the measured leaf sample	
*I*_sat_	Saturation light intensity corresponding to *J*_max_	μmol photons m^−2^ s^−1^
PSII	Photosystem II	
*k*_P_	Rate of pigment molecules from the excited state *k* to the ground state *i* due to photochemical reaction	s^−1^
*k*_D_	Rate of pigment molecules from the excited state *k* to the ground state *i* due to non-radiation heat dissipation	s^−1^
*α*	Initial slope of light–response curve of electron transport rate	μmol electron (μmol photon) ^−1^
*α* ^’^	Fraction of light absorbed by PSII	Dimensionless
*β* ^‘^	Leaf absorptance	Dimensionless
*β*	Photoinhibition coefficient	(μmol photons)^−1^ m^2^ s
*γ*	Light-saturated coefficient	(μmol photons)^−1^ m^2^ s
*ξ*_1_	Occupation probabilities of photochemistry	Dimensionless
*ξ*_2_	Occupation probabilities of non-radiation heat dissipation	Dimensionless
*ξ*_3_	Occupation probabilities of fluorescence	Dimensionless
*σ*_01_	Absorption cross-section of chlorophyll molecule from the ground state to the first excited state	m^2^
*σ*_1n_	Absorption cross-section of chlorophyll molecule from the first excited state to the other excited state	m^2^
*σ*_ik_	Eigen-absorption cross-section of photosynthetic pigment molecule from ground state *i* to excited state *k*	m^2^
*σ* ^’^_ik_	Effective optical absorption cross-section of photosynthetic pigment molecule from ground state *i* to excited state *k*	m^2^
*σ*_chl_	Absorption cross-section of chlorophyll pigment molecule	m^2^
*σ*_PSII_	Absorption cross-section of PSII	m^2^
*φ*	Use efficiency of excitons transport reaction center PSII to cause charge separation of P680	Dimensionless
*τ*	Average lifetime of the photosynthetic pigment molecules in the excited state *k*	S
*τ*_min_	Minimum average lifetime of photosynthetic pigment molecules in the excited state *k*	S

Currently, there are several methods or techniques for determining the values of these types of parameters. For example, Ley and Mauzerall (1982) estimated *σ*_chl_ in *Chlorella vulgaris* by measuring the rates of O_2_ production and found that ${\text{σ}}_{{\text{O}}_{2}}$ decreased with increasing growth light intensity. Surprisingly, there were no significant differences in *σ*_chl_ between cells grown under low-light or high-light conditions. De Boni et al. (2007) used the white light continuum (WLC) Z-scan technique to measure the resonant nonlinear spectrum of chlorophyll *a* extracted from *Spinacia oleracea*, to study *σ*_01_ (from the ground state to the first excited state), as well as *σ*_1n_ (from the first excited state to higher excited states), and showed that *σ*_01_ is dependent on wavelength. Based on the fast repetition rate (FRR) fluorescence technique, Suggett et al. (2007) found that as the growth light intensity increased, the *σ*_PSII_ and *σ*_chl_ in *Emiliania huxleyi* (Lohmann) decreased. Klughammer and Schreiber (2015) used a kinetic multi-color PAM chlorophyll fluorometer (referred to as MC-PAM method), which allows measurements of the sub-millisecond fluorescence rise kinetics upon the onset of strong variously colored actinic light and found that *σ*_PSII_ was a function of wavelength. Moreover, Ye et al. (2013a) proposed the YRS method, which can be used to determine *σ*_ik_ and *σ* ^′^_ik_ when the light–response curve of electron transport rate (*ETR–I* curve) and chlorophyll content are measured. In the results mentioned above, the dependence of *σ*_PSII_, *σ*_chl_, and *a*_L_*(λ) on wavelength has been confirmed (De Boni et al., 2007; Klughammer and Schreiber, 2015; Celebi-Ergin et al., 2022). Furthermore, previous studies have demonstrated that the *σ*_PSII_ and *σ*_chl_ values can vary during growth under different light intensities (Ley and Mauzerall, 1982; Suggett et al., 2007; Yang et al., 2018; Hu et al., 2021). In addition, Celebi-Ergin et al. (2022) found that the decrease in total chlorophyll resulted in an increased *a*_L_*(λ) when growth [CO_2_] increases. However, it is interesting to note that, despite variations among different photosynthetic organisms and environmental conditions, the magnitude of *σ*_chl_ [*σ*_ik_, *σ*_01_, and *a*_L_*(λ)] remains consistent at approximately 10^−21^ m^2^ (Ley and Mauzerall, 1982; De Boni et al., 2007; Suggett et al., 2007; Ye et al., 2016; Yang et al., 2018; Zuo et al., 2019; Hu et al., 2021; He et al., 2022). Only cell suspensions were used for the measurements mentioned above, except for the YRS method (2013a), which also measured leaves. The YRS is the only method capable of investigating *σ*_ik_ and *σ* ^′^_ik_ in the leaves of C_3_, C_4_, and CAM species under various conditions because it requires only a commercial portable gas-exchange and fluorescence systems and measured chlorophyll content (Ye et al., 2018; Zuo et al., 2019; Hu et al., 2021; He et al., 2022; Yang et al., 2023).

In order to address uncertainties about the relationship between light intensity, environmental factors (such as temperature, CO_2_, O_2_ and O_3_ concentrations), and the values of *σ*_chl_ or *σ*_PSII_, we have used here the YRS method to investigate *σ*_ik_ and *σ* ^′^_ik_ in plant leaves and a cyanobacterium in both dilute and dense suspensions. The objective of this study is to estimate both *σ*_ik_ and *σ* ^′^_ik_ of photosynthetic pigment molecules based on the experimental data collected on six different species grown under different conditions. Our results show that both *σ*_ik_ and *σ* ^′^_ik_ acclimate to growth light environment, CO_2_ concentration or different nitrogen source supplies, and their values are easily obtained by the YRS method independently of the species and experimental conditions.

## The mechanistic model of the *ETR–I* curve of Chl *a* fluorescence

*ETR* via PSII can be calculated with Eq. 1 (Ye et al., 2013a; Ye et al., 2013b) as:


(1) $$ETR=\frac{\alpha^{'}\beta^{'}N_{0}\sigma_{\text{ik}}\varphi }{S}\times \frac{1-\frac{\left(1-g_{\text{i}}/g_{\text{k}}\right)\sigma_{\text{ik}}\tau }{\xi_{3}+\left(\xi_{1}k_{\text{P}}+\xi_{2}k_{\text{D}}\right)\tau }I}{1+\frac{\left(1+g_{\text{i}}/g_{\text{k}}\right)\sigma_{\text{ik}}\tau }{\xi_{3}+\left(\xi_{1}k_{\text{P}}+\xi_{2}k_{\text{D}}\right)\tau }I}I$$

where *α* ^’^ is fraction of light absorbed by PSII (dimensionless), *β* ^’^ is leaf absorptance (dimensionless), *φ* is the exciton-use efficiency of PSII (dimensionless), *N*_0_ is total photosynthetic pigment molecules of the measured leaf sample, *S* is the measured area of leaf sample (m^2^), and *g*_i_ and *g*_k_ are the degeneracy of energy level of photosynthetic pigment molecules in the ground state (*i*) and excited state (*k*) (dimensionless), respectively. *k*_P_ and *k*_D_ are rates of the photochemical reaction and heat loss (s^−1^), respectively (Baker, 2008). *ξ*_1_, *ξ*_2_, and *ξ*_3_ were referred to as “statistical weight of photochemistry, heat loss, and fluorescence emission” (dimensionless), respectively, in our previous work (Ye, 2012; Ye et al., 2013a, Ye et al., 2013b). Furthermore, *σ*_ik_ (m^2^) is the eigen-absorption cross-section of photosynthetic pigment molecules from the ground state *i* to the excited state *k* (after light exposure), *τ* (s) is the average lifetime of the photosynthetic pigment molecules in the lowest excited state *k*, and *I* is the light intensity (μmol photons m^−2^ s^−1^). For the details of the derivation of the mechanistic model, see Ye et al. (2013a, b).

According to Ye et al. (2013a, b), *σ*_ik_, *τ*, *ξ*_1_, *ξ*_2_, *ξ*_3_, *g*_i_, *g*_k_, *k*_P_, and *k*_D_ (Eq. 1) are inherently specific but have different values, depending on the species and the environmental conditions (e.g., light, temperature, CO_2_ concentration, and the nutrient used). Therefore, for a given species and at a specific environmental condition, all parameters such as *σ*_ik_, *τ*, *ξ*_1_, *ξ*_2_, *ξ*_3_, *g*_i_, *g*_k_, *k*_P_, and *k*_D_ should be constant. In this case, we may assume that $\alpha =\frac{\alpha^{'}\beta^{'}N_{0}\sigma_{\text{ik}}\varphi }{S}$ [μmol electron (μmol photons)^−1^], which referred to as the initial slope of the *ETR–I* curve, $\beta =\frac{\left(1-g_{\text{i}}/g_{\text{k}}\right)\sigma_{\text{ik}}\tau }{\xi_{3}+\left(\xi_{1}k_{\text{P}}+\xi_{2}k_{\text{D}}\right)\tau }$ [(μmol photons)^−1^ m^2^ s], referred to as the “dynamic downregulation term of PSII/photoinhibition”, and $\gamma =\frac{\left(1+g_{\text{i}}/g_{\text{k}}\right)\sigma_{\text{ik}}\tau }{\xi_{3}+\left(\xi_{1}k_{\text{P}}+\xi_{2}k_{\text{D}}\right)\tau }$ [(μmol photons)^−1^ m^2^ s] referred to as “the saturation term of photosynthesis” in our previous work (Ye, 2012; Ye et al., 2013a, Ye et al., 2013b). Then, Eq. 1 can be simplified as:


(2) $$ETR=\alpha \frac{1-\beta I}{1+\gamma I}I.$$

Equations 1 and 2 describe the *ETR–I* function and characterize the interdependence between *ETR* and light intensity. Since Eq. 1 is a non-asymptotic function, it has the first derivative. When the first derivative of Eq. 1 equals zero, the saturation light intensity (*I*_sat_) corresponding to the maximum *ETR* can be calculated as follows:


(3) $$I_{\text{sat}}=\frac{\sqrt{\left(\beta +\gamma \right)/\beta }-1}{\gamma }.$$

Substituting Eq. 3 into Eq. 2, the maximum *ETR* (*ETR*_max_) can be calculated as:


(4) $$ETR_{\max}=\alpha {\left(\frac{\sqrt{\beta +\gamma }-\sqrt{\beta }}{\gamma }\right)}^{2}.$$

Moreover, when chlorophyll content (unit: mg m^−2^) is determined, *σ*_ik_ can be calculated as:


(5) $$\sigma_{\text{ik}}=\frac{S\alpha }{\alpha^{'}\beta^{'}\varphi N_{0}}=\frac{\alpha }{\alpha^{'}\beta^{'}\varphi n_{0}d}$$

where *n*_0_ is the number of photosynthetic pigment molecules of the measured leaf sample per unit volume (m^−3^), and *d* is the thickness of leaf sample (m). The biological significance of the other parameters in Eq. 5 is the same as that in Eq. 1.

In this work, we neither fit the values of the initial parameters of the model (*ξ*_i_, *k*_P_, *k*_D_, *τ*, and others) nor used their values assessed by other authors. We realize that the values of some of these parameters may differ significantly from the values known from the literature, and this is the weakness of the YRS model. Instead, we used Eq. 2 to fit the parameters *α*, *β*, and *γ* composed of the initial ones. We realize that this approach means using the YRS model actually as a phenomenological model. To some extent, this is due to the fact that the YRS model does not explicitly take into account the reactions of electron transport. Taking into account the reactions of electron transport and finding an interpretation of results of the fitting in terms of the values of the initial parameters of the model is the task for future research.

Equation 1 has been widely used in the literature to simulate the *ETR–I* curves of photosynthetic organisms and to extract several parameters from those curves (Serôdio et al., 2013; Morfopoulos et al., 2014; Sun et al., 2015; Ahammed et al., 2018; Robakowski et al., 2018; Yang et al., 2018; Zuo et al., 2019; Hu et al., 2021; He et al., 2022; Robakowski et al., 2022; Yang et al., 2023). In addition, when the chlorophyll content (mg m^−2^) is measured, besides *α*, *ETR*_max_, and *I*_sat_, some other key parameters characterizing photosynthetic pigment molecules (e.g., *σ* ^′^_ik_; the minimum average life time of the light-harvesting pigment molecules in the lowest excited state, *τ*_min_; the light-harvesting pigment molecules in the excited state, *N*_k_) can also be obtained (Ye et al., 2013a; Ye et al., 2013b).

## Effective absorption cross-section of photosynthetic pigment molecules

The *σ* ^′^_ik_, based on uniform light absorption in leaves, can be described as follows (Ye et al., 2013a; Ye et al., 2013b):


(6a) $$\sigma_{\text{ik}}^{'}=\frac{\sigma_{\text{ik}}}{1+\frac{\left(1+g_{\text{i}}/g_{\text{k}}\right)\sigma_{\text{ik}}\tau I}{\xi_{3}+\left(\xi_{1}k_{\text{P}}+\xi_{2}k_{\text{D}}\right)\tau }}\left[1-\frac{\left(1-g_{\text{i}}/g_{\text{k}}\right)\sigma_{\text{ik}}\tau I}{\xi_{3}+\left(\xi_{1}k_{\text{P}}+\xi_{2}k_{\text{D}}\right)\tau }\right]$$

Or


(6b) $$\sigma_{\text{ik}}^{'}=\frac{1-\beta I}{1+\gamma I}\sigma_{\text{ik}}$$

The biological significance of the parameters in Eq. 6 is the same as that in Eq. 1. We note, from Eq. 6, that *σ* ^′^_ik_ will decrease with increasing *I* when photosynthetic organisms are under certain environmental conditions; from Eq. 6, *σ* ^′^_ik_ = *σ*_ik_ when *I* = 0 μmol photons m^−2^ s^−1^. As such, the *σ* ^′^_ik_ is not a constant under any given light intensity (except when *I* = 0 μmol photons m^−2^ s^−1^).

## Data processing and statistical analysis

The mechanistic model presented above was fitted to obtain key parameters (e.g., *ETR*_max_, *I*_sat_ *, σ*_ik_, and *σ* ^′^_ik_) defining the *ETR–I* curves, using the Photosynthesis Model Simulation Software (PMSS) (http://photosynthetic.sinaapp.com, Jinggangshan University, Ji’an, China). When fitting the *ETR–I* curves using the YRS model, the initial values are set as follows: *α*_e_ = 0.06, *β*_e_ = 0.002, *γ*_e_ = 0.01.

All statistical tests were performed using the statistical package SPSS *18.5* statistical software (SPSS, Chicago, IL). Student’s *t*-test was conducted to test whether there were significant differences between the fitted and the measured values of quantitative traits (such as *ETR*_max_ and *I*_sat_). Goodness of the fit of experimental observations with results obtained with the mathematical model was assessed using the coefficient of determination (*R* ^2 ^= 1 – SSE/SST, where SST is the total sum of squares and SSE is the error sum of squares).

## Examples of model application

**Figure 1:**
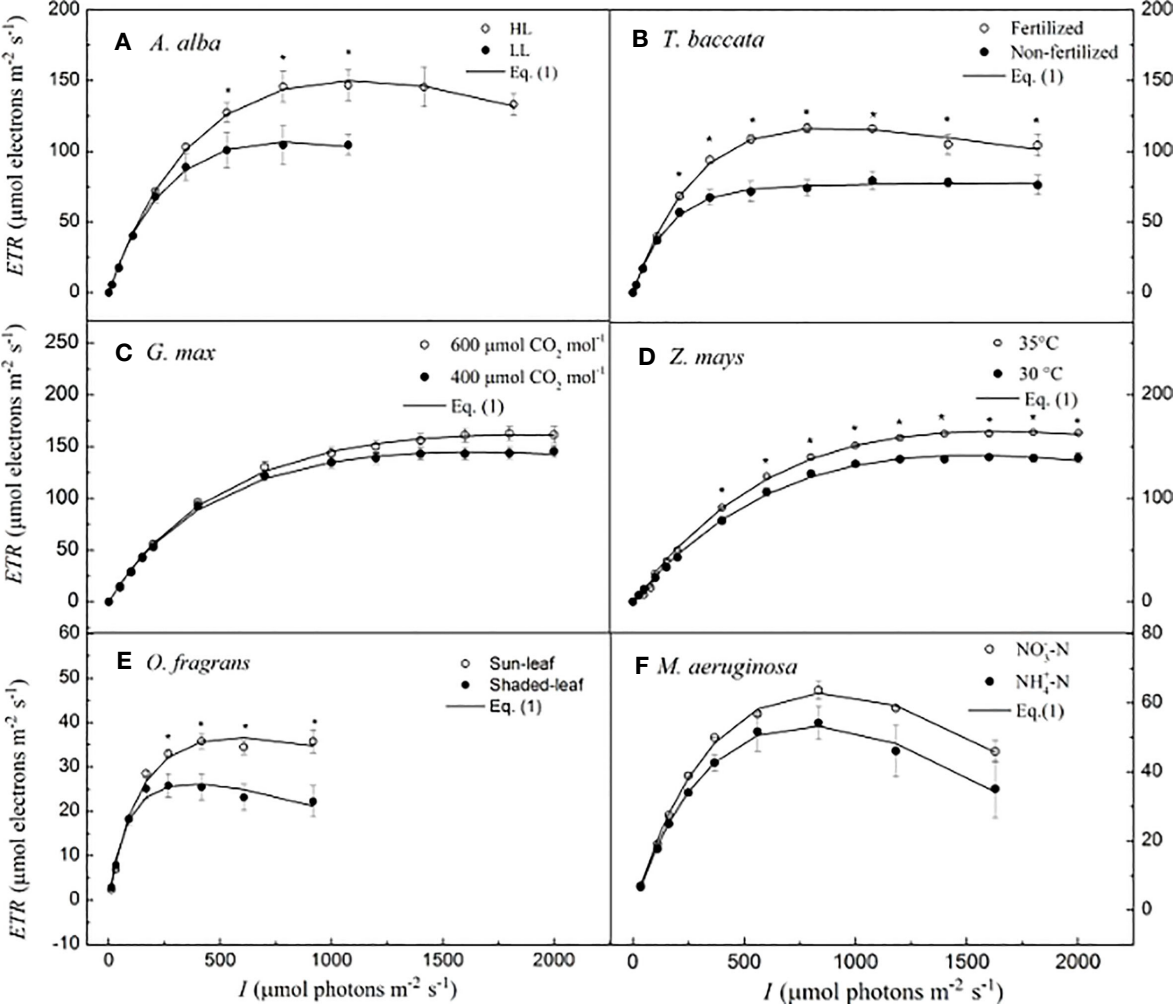
Light-response curves of photosynthetic electron flow and curves fitted by Eq. I for six species. **(A)** for *A. alba*; **(B)** for *T. baccata*; **(C)** for *G. max*; **(D)** for *Z. mays*; **(E)** for *O. fragrans*; and **(F)** for *M aeruginosa*. An asterisk indicates that mean values of ETR at the given / value are significantly different using Student's t-test at p<0.05 (n-5-12). Data are mean±SE (n-3-12). The results of the statistical analyses, see **Table 2**. I, lightintensity.

Details of the growth conditions, the species examined, and the procedures for generating the *ETR–I* curves to be examined with our model are described in the Supporting Information [for *A. alba*, see Robakowski et al., 2022; for *T. baccata*, see Robakowski et al., 2018; for *G. max* at 400 and 600 μmol CO_2_ mol^−1^, see Ye et al., 2018; for *Z. mays* at 30°C and 35°C temperatures, see Wang et al., 2022; for *O. fragrans* (both for sun-leaf and shaded-leaf), see Hu et al., 2021; and for *M. aeruginosa*, under two different nitrogen supplies, see Yang et al., 2023]. In the present study, representative *ETR–I* curves (fitting the model of Eq. 1) for four C_3_ species (i.e., *A. alba*, *T. baccata*, *G. max*, and *O. fragrans*), one C_4_ species (*Z. mays*), and one cyanobacterium (*M. aeruginosa*) are shown in **Figure 1**. For *A. alba* grown under HL (**Figure 1A**), *O. fragrans* (shaded-leaf) (**Figure 1E**) and *M. aeruginosa* grown under two nitrogen supplies (**Figure 1F**), the value of *ETR* initially increased (almost linearly) with the light intensity towards saturation, and subsequently, beyond the highest irradiance (i.e., *I*_sat_), it showed a significant degree of decline, likely reflecting an onset of dynamic downregulation of PSII, especially in *M. aeruginosa*. On the other hand, *A. alba* grown under LL (**Figure 1A**), *T. baccata* (fertilization) (**Figure 1B**), *G. max* at 400 μmol CO_2_ mol^−1^ under shaded conditions (**Figure 1C**), *Z. mays* (**Figure 1D**), and *O. fragrans* (sun-leaf) (**Figure 1E**) exhibited only a small decline of *ETR* with increasing light intensity beyond the *I*_sat_. Data for *T. baccata* (non-fertilization) (**Figure 1B**) and *G. max* at 600 μmol CO_2_ mol^−1^ under shaded conditions (**Figure 1C**) showed that *ETR* hardly increases with increasing light intensity beyond the *I*_sat_. Moreover, the fitted curves show that Eq. 1 reproduces quite well the *ETR–I* curves of all the six species, regardless of whether there is dynamic downregulation, and this with extremely good fits (*R* ^2^ ≥ 0.995) (**Figure 1**; **Table 2**).

**Table 2:** Results fitted by Eq. 1 and observation values of photosynthetic parameters for six species under various conditions (mean ± SE, *n* = 3-12).

	*A. alba*							*T. baccata*							*G. max*				
	LL			HL				Fertilization			Non-fertilization				400 μmol mol^–1^			600 μmol mol^–1^	
	Eq. 1	Obs.		Eq. 1		Obs.		Eq. 1	Obs.		Eq. 1			Obs.	Eq. 1		Obs.	Eq. 1	Obs.
*α*	0.502 ±0.016	–		0.499 ± 0.009		–		0.537 ± 0.009	–		0.579 ± 0.014			–	0.356 ± 0.006a		–	0.356 ± 0.002a	–
*I*_sat_	836.83 ± 55.81^a^	977.67 ± 97.33^a^		1,061.60 ± 33.21^a^		1,301.67± 133.33^a^		1,150.91 ± 132.92^a^	1,224.08 ± 127.99^a^		1,376.31 ± 342.52^a^			1,215.58 ± 128.11^a^	1,647.45 ± 76.60^a^		1,601.59 ± 0.64^a^	1,930.19 ± 73.54^a^	1,999.82 ± 0.69^a^
*J*_max_	107.55 ± 7.32^a^	107.04 ± 6.63a		137.56 ± 8.69^a^		136.68 ± 7.49^a^		111.26 ± 3.86^a^	110.62 ± 4.34^a^		81.12 ± 4.93^a^			81.58 ± 4.87^a^	144.64 ± 5.72^a^		143.51 ± 5.21^a^	162.07 ± 5.92^a^	162.38 ± 8.08a
*σ*_ik_ (×10^−21^)	1.25 ± 0.05^B^	–		1.65 ± 0.03^A^		–		1.44 ± 0.11^A^	–		1.37 ± 0.14^A^			–	3.92 ± 0.07^A^		–	3.91 ± 0.05^A^	–
Chlorophyll content (mg m^−2^)	1,582.34 ± 9.12^A^			1,194.08 ± 13.58^B^				1,603.86 ± 138.48^A^			1,900.21 ± 218.99^A^				900.40 ± 12.25			900.40 ± 12.25	
*R* ^2^	0.998	–		0.998		–		0.995	–		0.996			–	0.996		–	0.999	–
	*Z. mays*							*O. fragrans*							*M. aeruginosa*				
	30°C			35°C				Shaded-leaf			Sun-leaf				NO^-^_3_-N			NH_4_^+^-N	
	Eq. 1	Obs.		Eq. 1		Obs.		Eq. 1	Obs.		Eq. 1			Obs.	Eq. 1		Obs.	Eq. 1	Obs.
*α*	0.269 ± 0.021	–		0.282 ± 0.012		–		0.282 ± 0.012	–		0.295 ± 0.012			–	0.222 ± 0.012		–	0.205 ± 0.003	–
*I*_sat_	1,539.44 ± 66.23^a^	1,666.67 ± 176.38^a^		1,665.35 ± 154.57^a^		1,600.01 ± 230.94^a^		384.47 ± 39.83^a^	306.60 ± 48.25^a^		482.45 ± 26.21^a^			717.20 ± 122.96^a^	792.32 ± 75.88^a^		833.00 ± 0.00a	869.80 ± 19.46^a^	833.00 ± 0.00^a^
*J*_max_	142.71 ± 2.27^a^	142.10 ± 3.95^a^		179.55 ± 8.72^a^		179.88 ± 8.45^a^		26.43 ± 2.92^a^	26.50 ± 2.76^a^		36.32 ± 1.45^a^			37.75 ± 2.23^a^	62.99 ± 1.63^a^		63.67 ± 2.64a	53.73 ± 5.70^a^	54.20 ± 4.77^a^
*σ*_ik_ (×10^−21^)	2.47 ± 0.20^A^	–		2.26 ± 0.15^A^		–		4.66 ± 0.15^B^	–		5.68 ± 0.25^A^			–	3.56 ± 0.21^B^		–	5.06 ± 0.11^A^	–
Chlorophyll content (mg m^−2^)	432.32 ± 7.6			432.32 ± 7.6				342.30 ± 21.66^A^			210.60 ± 20.52^B^				247.19 ± 3.31^A^			159.97 ± 1.37^B^	
*R* ^2^	0.999	–		0.999		–		0.995	–		0.996			–	0.995		–	0.996	–

α, initial slope of ETR–I curves [μmol electron (μmol photon)^−1^]; I_sat_, saturation irradiance (μmol photons m^−2^s^−1^); J_max_, the maximum electron transport rate (μmol electrons m^−2^s^−1^); σ_ik_, eigen-absorption cross-section of photosynthetic pigment molecules (m^2^); R^2^, determination coefficient. The different superscript letters followed by the values are significantly different between fitted values and observation values within the same species using Student’s t-test at p< 0.05 (n = 3–12). For σ_ik_ and chlorophyll content, the different superscript capital letters followed by the values are significantly different between fitted values and observation values for the same species with different treatments using Student’s t-test at p< 0.05 (n = 3–12).

**Table 2** lists the photosynthetic parameters (such as *α*, *ETR*_max_, and *I*_sat_) estimated by Eq. 1 and their corresponding observed values for the four C_3_ species, a C_4_ species, and a cyanobacterium grown under different conditions, described earlier. In this study, we did not observe significant differences between the estimated values of *ETR*_max_ (and *I*_sat_) and their corresponding observed values (*p* > 0.05). Furthermore, **Table 2** shows that the values of *σ*_ik_, calculated by Eq. 5, have different values ranging from 1.25 × 10^−21^ m^2^ to 5.68 ×10^−21^ m^2^ for the six species used in this study. For *A. alba*, there was a significant difference between *σ*_ik_ at HL and at LL (*p<* 0.05), with the values of *σ*_ik_ at HL being higher than that at LL; for *O. fragrans*, there was a significant difference between *σ*_ik_ evaluated for the shaded-leaf and for the sun-leaf (*p<* 0.05), with the values of *σ*_ik_ of the sun-leaf being larger than that of the shaded-leaf; for *M. aeruginosa*, there was a significant difference between *σ*_ik_ under NO_3_^−^-N supply and under NH_4_^+^-N supply (*p<* 0.05), with the values of *σ*_ik_ under NO_3_^−^-N supply being smaller than that under NH_4_^+^-N supply. In addition, no significant difference was found between *σ*_ik_ evaluated in *G. max* at 400 μmol CO_2_ mol^−1^ and 600 μmol CO_2_ mol^−1^; furthermore, no significant difference was found for *σ*_ik_ between *Z. mays* at 30°C and 35°C. Also there were no significant differences in *σ*_ik_ between *T. baccata* seedlings grown in fertilization and in non-fertilization conditions.

## Effective absorption cross-section of photosynthetic pigment molecules

**Figure 2:**
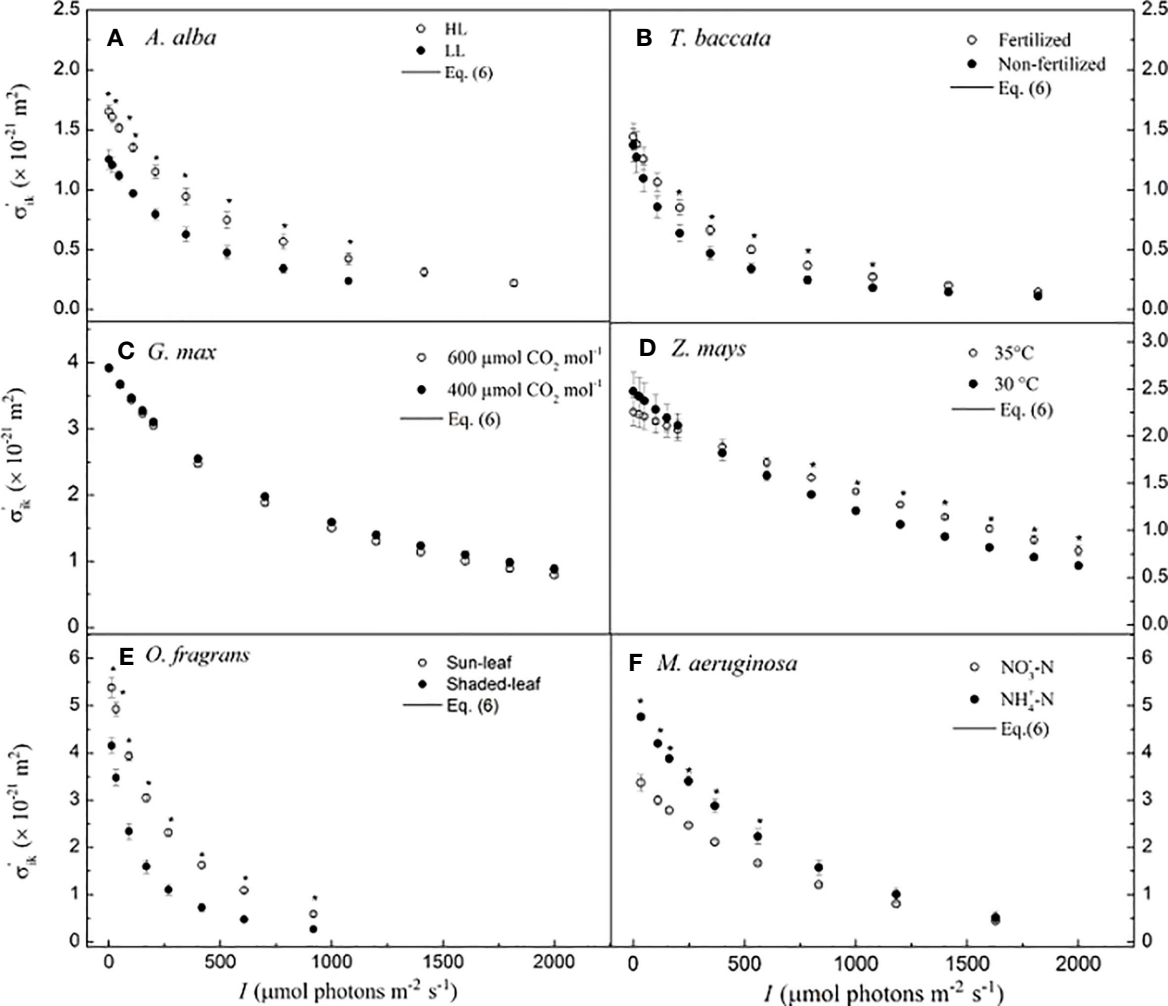
Light-response curves of the effective absorption cross-section of photosynthetic pigment molecules calculated by Eq. 6 for six species. **(A)** for *A. alba*; **(B)** for *T. baccata*; **(C)** for *G. max*; **(D)** for *Z. mays*; **(E)** for *O. fragrans*; and **(F)** for *M aeruginosa*. An asterisk indicates that mean values of σ'ik at the given I value are significantly different using Student's t-test at p< 0.05 (n = 3-12). Data are mean ± SE (n=3-12). I, light intensity.

The values of *σ* ^′^_ik_ nonlinearly decrease with *I* in *A. alba* at HL and LL (**Figure 2A**), in *T. baccata* grown under fertilized and non-fertilized conditions (**Figure 2B**), in *G. max* at 400 μmol CO_2_ mol^−1^ and 600 μmol CO_2_ mol^−1^ (**Figure 2C**), in *O. fragrans* of sun-leaf and shaded-leaf (**Figure 2E**), and in *M. aeruginosa* grown under NO_3_^−^-N supply and NH_4_^+^-N supply (**Figure 2F**). For *Z. mays* at 30°C versus that at 35°C (**Figure 2D**), we observed that *σ* ^′^_ik_ decreases almost nonlinearly with increasing *I*. In addition, for *A. alba*, there is a significant difference between *σ* ^′^_ik_ at HL and at LL for any *I* (*p<* 0.05) (**Figure 2A**), and the values of *σ* ^′^_ik_ at HL are higher than those of *σ* ^′^_ik_ at LL. For example, at 783 μmol photons m^−2^ s^−1^ for *A. alba*, the value of *σ* ^′^_ik_ at HL is 0.697 (± 0.060) × 10^−21^ m^2^, while the value of *σ* ^′^_ik_ at LL is 0.263 (± 0.032) × 10^−21^ m^2^. For *Z. mays*, there is a significant difference between *σ* ^′^_ik_ at 30°C and at 35°C (*p<* 0.05) when the light intensity ranges from 800 to 1,800 μmol photons m^−2^ s^−1^, with the *σ* ^′^_ik_ values at 35°C higher than those at 30°C. For instance, at 1,800 μmol photons m^−2^ s^−1^ for *Z. mays*, the value of *σ* ^′^_ik_ at 35°C is 0.898 (± 0.049) × 10^−21^ m^2^, while the value of *σ* ^′^_ik_ at 30°C is 0.717 (± 0.030) × 10^−21^ m^2^. For *O. fragrans*, there is a significant difference between *σ* ^′^_ik_ for shaded-leaf and sun-leaf (*p<* 0.05), as the values of *σ* ^′^_ik_ of sun-leaf are higher than those of shaded-leaf at any light intensity. For instance, at 606 μmol photons m^−2^ s^−1^ for *O. fragrans*, the value of *σ* ^′^_ik_ for the sun-leaf is 0.588 (± 0.044) × 10^−21^ m^2^, while the value of *σ* ^′^_ik_ for the shaded-leaf is 0.266 (± 0.043) × 10^−21^ m^2^. For *M. aeruginosa*, there is a significant difference between *σ* ^′^_ik_ under NO_3_^−^-N supply and under NH_4_^+^-N supply (*p<* 0.05) when the light intensity is below 559 μmol photons m^−2^ s^−1^, and in this case, the values of *σ* ^′^_ik_ under NO_3_^−^-N supply are less than those under NH_4_^+^-N supply. For instance, at 559 μmol photons m^−2^ s^−1^, for *M. aeruginosa*, the value of *σ* ^′^_ik_ is 1.666 (± 0.037) × 10^−21^ m^2^ under NO_3_^−^-N supply, and the value of *σ* ^′^_ik_ is 2.230 (± 0.162) × 10^−21^ m^2^ under NH_4_^+^-N supply. In contrast, for *G. max*, no significant difference between *σ* ^′^_ik_ at 400 μmol CO_2_ mol^−1^ and at 600 μmol CO_2_ mol^−1^ is found (**Figure 2C**).

## Discussion

We have simulated the *ETR−I* curves of six different species under different conditions using the mechanistic model developed by Ye et al. (2013a, b). Fitting this model to previously collected data (including HL and LL conditions, fertilized and non-fertilized conditions, two CO_2_ concentrations, two temperatures, and different species, i.e., four C_3_, one C_4_, and one cyanobacterium) showed extremely good fits (*R* ^2^ ≥ 0.995), and the fitted values for *ETR*_max_ and *I*_sat_ were consistent with all the observations (**Table 2**), and with no significant difference between the fitted values for *ETR*_max_ (and *I*_sat_) and their corresponding observations (*p<* 0.05; **Table 2**). This shows that Eq. 1 is highly adequate for fitting *ETR−I* curves regardless of dynamic downregulation of PSII/photoinhibition in the photosynthetic organisms used in our current study. Furthermore, our results are consistent with previous studies (Ye et al., 2013a; Ye et al., 2013b; Ye et al., 2016; Yang et al., 2018; Zuo et al., 2019; Hu et al., 2021; He et al., 2022).

There are several methods that can be used to determine *σ*_chl_. For example, Ley and Mauzerall (1982) estimated *σ*_PSII_ of *C. vulgaris* by measuring the rates of O_2_ production and found that the estimated value of *σ*_chl_ was 2.90 (± 0.10) × 10^−21^ m^2^. At the same time, these authors found no significant differences in *σ*_chl_ measured on LL-grown and HL-grown cells. After 25 years of Ley and Mauzerall’s work, De Boni et al. (2007) used WLC Z-scan technique to study the values of *σ*_01_ and *σ*_1n_ of Chl *a* and found that the value of *σ*_01_ was ~4.7 × 10^−21^ m^2^ at 680 nm, while the value of *σ*_1n_ was ~0 m^2^. Furthermore, De Boni et al. (2007) found that *σ*_01_ is wavelength-dependent, while Klughammer and Schreiber (2015) using a MC-PAM method determined that *σ*_chl_ is a function of wavelength and can be determined in combination with chlorophyll content. Moreover, Suggett et al. (2007) using the fast repetition rate fluorescence technique studied that both *σ*_PSII_ and *σ*_chl_ in *E. huxleyi* decrease as the growth light intensity increases. For instance, *σ*_chl_ was 3.25 × 10^−21^ m^2^ in cells grown under low light (25 μmol photons m^−2^ s^−1^), but decreased to 2.95 × 10^−21^ m^2^ under high light (600 μmol photons m^−2^ s^−1^). Their results revealed that growth light intensity affects the values of *σ*_chl_, and indirectly demonstrated that *σ*_chl_ is a function of light intensity, and seems to decrease with light intensities. Thus, although the values of *σ*_chl_ obtained with various methods are different, the estimated values are in the same order of magnitude (approximately 10^−21^ m^2^). These results are consistent with our results given in **Table 2**.

However, *σ*_chl_, *σ*_01_, *a*_L_*(λ), and *σ*_ik_ have different definitions and different biological significance. For example, if *i* = 0 and *k* = 1, *σ*_ik_ represents the chlorophyll molecules that are excited from the ground state (*i* = 0) to the first excited state (*k* = 1), i.e., *σ*_01_. In addition, when *k* ≥ 1, *σ*_ik_ represents the average light absorption cross-section of a photosynthetic pigment since the accessory pigments transfer the absorbed solar energy to chlorophyll molecules, which will make a transition from the ground state (*i* = 0) to different excited states (*k* ≥ 1), depending on the wavelength of light. It, thus, follows that *σ*_ik_ has the same biological significance as *σ*_chl_ and *a*_L_*(λ). Consequently, both *σ*_chl_ and *a*_L_*(λ) indicate the average light absorption cross-section of the photosynthetic pigment under light illumination.

From an operational perspective of the YRS method (Ye et al., 2013a, Ye et al., 2013b), the value of *σ*_ik_ can be easily estimated by using Eq. 5, for any species and under different environmental conditions. For instance, the value of *σ*_ik_ is 1.12 × 10^−21^ m^2^ for *T. baccata* (fertilization), while it is 5.68 × 10^−21^ m^2^ for *O. fragrans*. In this study, the values of *σ*_ik_ for other photosynthetic organisms were found to be between these two values (**Table 2**). Thus, our work demonstrates that although the experimental materials and methods used have been different (Ley and Mauzerall, 1982; De Boni et al., 2007; Suggett et al., 2007; Ye et al., 2013a, Ye et al., 2013b; Klughammer and Schreiber, 2015; Celebi-Ergin et al., 2022), the values of *σ*_ik_ or *σ*_chl_ are approximately of the same order of magnitude (approximately 10^−21^ m^2^). Previous studies have demonstrated that the relationship between *σ*_chl_ and light adaptation is crucial for understanding how plants and algae adapt to different light environments (Ley and Mauzerall, 1982; Suggett et al., 2007; Neale and Melis, 2010). Plant species and algae grown under different light conditions may have different evolutionary strategies in optimizing light absorption and utilization (Neale and Melis, 2010; Buckley and Diaz-Espejo, 2015). The magnitude of *σ*_chl_ can reflect the light-harvesting capability and light adaptation ability of plants and algae. In this study, the value of *σ*_ik_ for *A. alba* grown under LL conditions is smaller than that of *σ*_ik_ for *A. alba* grown under HL (**Table 2**), and there is a significant difference between them. This result is different from the results obtained by Ley and Mauzerall (1982) and Suggett et al. (2007). Ley and Mauzerall (1982) found that there was no significant difference between *σ*_chl_ of *C. vulgaris* in LL and in HL, while Suggett et al. (2007) found that *σ*_chl_ of *E. huxleyi* (B11) at LL growth was higher than those under HL growth. This indicates that the shade-tolerant conifer *A. alba* has a different strategy from the alga *E. huxleyi* grown under different light environments. This inconsistency between our results and those of others may reflect evolutionary differences between the terrestrial plants and algae, which are adapted to totally different light environments. In addition, in our present study, we found that there was no significant difference between *σ*_ik_ values at 30°C and 35°C for *Z. mays*, or at 400 μmol CO_2_ mol^−1^ and 600 μmol CO_2_ mol^−1^ for *G. max*. These results are inconsistent with those obtained by Celebi-Ergin et al. (2022) on eelgrass (*Zostera marina* L.), as they found that the decrease in total chlorophyll at greater [CO_2_] values resulted in an increased *a*_L_*(Λ). It is uncertain whether this difference between our results and those of the other authors result from the different experimental conditions or the species-specific, genetic features, and thus further research is needed.

Suggett et al. (2007) found that *σ*_chl_ of *E. huxleyi* (B11) decreased as the growth light intensity increased. Their results showed that *E. huxleyi* (B11) at the lowest growing irradiance (25 μmol photons m^−2^ s^−1^) had the highest values of *σ*_chl_ (i.e., 3.25 × 10^−21^ m^2^), while at the highest light intensity (600 μmol photons m^−2^ s^−1^), it had the smallest values of *σ*_chl_ (i.e., 2.95 × 10^−21^ m^2^). For the intermediate growth irradiances (i.e., 150 and 300 μmol photons m^−2^ s^−1^), the values of *σ*_chl_ were between 2.95 × 10^−21^ m^2^ and 3.25 × 10^−21^ m^2^. These results suggest that the value of *σ*_chl_ is a function of light intensity, and that it decreases with light intensity. Therefore, the results of Suggett et al. (2007) are consistent with our conclusion on *σ* ^′^_ik_, which decreases nonlinearly with increasing *I*. However, Ley and Mauzerall (1982) found no significant differences in *σ*_chl_ measured on LL-grown and HL-grown cells. Moreover, Klughammer and Schreiber (2015) found that *σ*_PSII_ (625 nm) nonlinearly increases with increasing light, and then it decreases nonlinearly at high light intensities, but the decrease in *σ*_PSII_ (625 nm) with light intensity depends on the value of *J*_p_ (where *J*_p_ is a parameter characterizing the energetic connectivity between PSII units; see Stirbet, 2013). For instance, Klughammer and Schreiber (2015) reported that *σ*_PSII_ (625 nm) nonlinearly increases with *I* beyond 2,000 μmol photons m^−2^ s^−1^ for *J*_p_ = 2. This obviously contradicts our common sense expectation, since photoinhibition would occur in most dilute suspensions of *Chlorella* exposed to high light intensities of over 2,000 μmol photons m^−2^ s^−1^.

We emphasize that our results have been derived from instantaneous measurement of *ETR–I* curves of plants and one cyanobacterium; thus, some differences between *σ*_chl_ obtained by Ley and Mauzerall (1982) and by Suggett et al. (2007) and *σ* ^′^_ik_ may result from the different growth irradiance conditions. Compared to the complex methods and techniques required for obtaining parameters such as *σ*_PSII_, *σ*_chl_, and *a*_L_*(λ) in Ley and Mauzerall (1982); Suggett et al. (2007); De Boni et al. (2007); Klughammer and Schreiber (2015), and Celebi-Ergin et al. (2022), we can easily determine the values of *σ* ^′^_ik_ by Eq. 6 for any given species under any environmental condition. In addition, it should be noted that due to Eq. 1 being a non-asymptotic function, it possesses a first derivative. According to the principles of calculus, when the first derivative of Eq. 1 equals zero, *I*_sat_ and its corresponding *ETR*_max_ can be calculated using Eqs. (4) and (3), respectively. However, it is only when the selected model perfectly fits the *ETR*–*I* curve that the fitted values will match the observed values. Empirical models such as the single exponential model, double exponential model, non-rectangular hyperbolic model, and Suggett et al.’s semi-mechanistic model are asymptotic and cannot accurately fit the *ETR*–*I* curve when plants or algae experience dynamic downregulation of PSII/photoinhibition (Platt et al., 1980; Suggett et al., 2007; Brading et al., 2011; Yang et al., 2023). As a result, obtaining *ETR*_max_ and *I*_sat_ becomes challenging, let alone other parameters reflecting the intrinsic characteristics of photosynthetic pigment molecules (such as *σ*_ik_, *σ* ^′^_ik_, *τ*_min_, and *N*_k_). Although the double exponential model can be used to fit the *ETR*–*I* curve of algae, it needs to be coupled with modified exponential model to derive *ETR*_max_ and *I*_sat_, and the obtained *ETR*_max_ and *I*_sat_ values are significantly higher than the measured values (Maxwell and Johnson, 2000; Brading et al., 2011; Buckley and Diaz-Espejo, 2015). On the other hand, the YRS method not only effectively fits the *ETR* response curves of plants and algae to light but also shows no significant difference between the fitted *ETR*_max_ and *I*_sat_ values and the observation values (Liang et al., 2018; Yang et al., 2018; Furutani et al., 2022). In this study, the results obtained using the YRS model for fitting also exhibit no significant difference from the observation values, further demonstrating the applicability and reliability of this model.

Furthermore, the reason why *σ* ^′^_ik_ decreases with light intensity in our study, in addition to the characteristics of harvesting pigment molecule itself (e.g., change in *σ*_ik_, *ξ*_1_, *ξ*_2_, *ξ*_3_, *g*_i_, and *g*_k_), may be due to the photo-relocation movement of chloroplasts to anticlinal cell walls (i.e., negative blue/UV-A light-dependent chloroplast phototaxis) where light absorption is reduced (Kasahara et al., 2002; Lazár et al., 2022), presumably to minimize the saturation of photosynthesis by decreasing the amount of excess excitation energy (Murchie and Niyogi, 2011). Such observations may help to investigate the underlying nature of plants’ photoprotection mechanism under high light intensity (Murchie and Niyogi, 2011), since decreases in *σ* ^′^_ik_ with light intensity would prevent the pigment molecules from absorbing light energy further, and could thus provide us a means to quantitatively investigate the photoinhibition and photoprotection in photosynthetic organisms.

Although different fluorescence and O_2_ evolution methods, such as the specific O_2_ evolution technique (Ley and Mauzerall, 1982; Celebi-Ergin et al., 2022), WLCZ-scan technique (De Boni et al., 2007), and FRR technique (Suggett et al., 2007), may estimate the light absorption of chlorophyll pigments, these methods have the disadvantage to be technically challenging. Moreover, they use only dilute suspensions of cells, which is why these methods have not been widely applied for the *σ*_chl_ evaluation. On the other hand, while the MC-PAM technique (Klughammer and Schreiber, 2015) can be used not only for dense suspensions of unicellular algae, but also for optically more complex samples (like leaves and corals), there still remains a difficulty in determining *σ*_chl_, due to the complexity of their methods used for data processing. In contrast with the above approaches, combined commercial portable gas-exchange and fluorescence systems (e.g., LI-6400, Li-Cor Inc., Lincoln, Nebraska, USA; CIRAS-II, PP Systems, Hitchin, UK; LCA4 ADC-Biosciences, Hoddesdon, UK; Dual-PAM-100, Walz Inc., Germany), the values of *σ*_ik_ can be obtained easily by using the YPS method presented here for any plant species, algae, and cyanobacteria, under diverse environmental conditions, once their chlorophyll content is measured. Therefore, our method has a greater advantage over the methods and techniques in determining the values of *σ*_ik_ (see Eq. 5).

In conclusion, (1) the mechanistic model, used in this research, has universality in fitting *ETR−I* curves and in estimating photosynthetic parameters such as *ETR*_max_, *I*_sat_, *σ*_ik_, and *σ* ^′^_ik_; (2) compared with other methods, estimating *σ*_chl_, *σ*_PSII_(λ), or *a*_L_*(λ), our method is much more convenient in determining *σ*_ik_ and *σ* ^′^_ik_ than those used by other authors (Ley and Mauzerall, 1982; De Boni et al., 2007; Suggett et al., 2007; Klughammer and Schreiber, 2015; Celebi-Ergin et al., 2022); (3) Eqs. 5 and 6 can be used to investigate the effects of different environmental factors (e.g., CO_2_, O_2_, O_3_, NO_2_, temperature, and nutrition) on *σ*_ik_ and *σ* ^′^_ik_, respectively. We conclude that Eq. 1 (see above for the description of the YRS method) provides a powerful means to predict and simulate the *ETR−I* curves, and to obtain several important parameters such as *ETR*_max_, *I*_sat_, *σ*_ik_, and *σ* ^′^_ik_. Furthermore, the latter can be used to investigate light energy absorption properties of photosynthetic pigment molecules and to understand how plants, algae, and cyanobacteria modify their light-harvesting properties underlying photo-acclimation, photoprotection, and abiotic stress. Dynamic changes of *σ* ^′^_ik_ in response to light indicate that these pigment–protein complexes are used by the photosynthetic organisms, not only as antennae capturing light energy, but also to serve an important role in light energy partitioning and dissipation. Thus, we recommend the use of the YRS method to calculate the values of *σ*_ik_ and *σ* ^′^_ik_ to investigate light energy absorption properties of photosynthetic pigment molecules for all plants, algae, and cyanobacteria under any environmental condition. We are planning further studies (i) for evaluating the performance of Eq. 1 when applied to observations on different photosynthesizing organisms under extreme environmental conditions; (ii) for estimating *σ*_ik_ and *σ* ^′^_ik_ when the samples, under investigation, are concurrently stressed by various environmental factors, including, e.g., low or high temperatures, different CO_2_, O_2_, O_3_, and NO_2_ concentrations, water stress, or salt stress; and (iii) for comparison of *σ*_ik_ and *σ* ^′^_ik_ among different functional groups of plants, cyanobacteria, and algae, to better understand evolutionary mechanisms of adaptation in photosynthetic organisms to different environmental conditions.

In order to facilitate others to utilize our developed mechanistic model of *ETR−I*, we have developed and exploited PMSS (http://photosynthetic.sinaapp.com). Using measured *ETR−I* data and combining it with measurements on chlorophyll content (mg m^−2^), many characteristics, associated with light energy absorption of chlorophyll molecules, can be obtained with this software.

## Data availability statement

The original contributions presented in the study are included in the article/**Supplementary Material**. Further inquiries can be directed to the corresponding authors.

## Author contributions

All authors contributed to the conception of the work. Z-PY and X-LY mainly fitted the data. Z-PY and F-BW drafted the original manuscript. All authors critically reviewed and revised the manuscript with new data sets and contributed substantially to the completion of the present study. All authors contributed to the article and approved the submitted version.
