# Supplementary material for: Investigation on absorption cross-section of photosynthetic pigment molecules based on a mechanistic model of the photosynthetic electron flow-light response in C3, C4 species and cyanobacteria grown under various conditions
Source: Front Plant Sci. 2023 Aug 29;14:1234462. doi: 10.3389/fpls.2023.1234462 (PMC10497745; doi:10.3389/fpls.2023.1234462)
Supplement: Supplementary file 1 [file DataSheet_1.pdf]

Supporting Information

Materials and methods

The six different species that used for the experimental data collection are:

(**i**) *Abies alba* Mill. with a carboxylation pathway of C_3 grown at high light (HL, 100% of full sun irradiation) and low light (LL, 40% of full sun irradiation) in Miedzylesie in Poland (Robakowski et al., 2022). Chlorophyll *a* fluorescence was measured with a fluorescence monitoring system (FMS 2, Hansatech, Norfolk, UK). In August 2020, full expanded current-year needles were dark-adapted at room temperature (21-23 ℃) for 30 min. The modulated and saturated light intensity used for chlorophyll *a* fluorescence measurements are set at 0.05 μmol m^{-2} s^{-1} and 15.3 mmol m^{-2} s^{-1}, respectively. Other parameters are set according to Robakowski et al., 2022. The *ETR* were calculated as Maxwell et al. (2000) using the formula $ETR = \alpha \times \Phi_{PSII} \times PPF \times 0.5$ (α, needle absorptance; Φ_{PSII}, PSII quantum yield; *PPF*, the mean relative value of photosynthetic).

(**ii**) Seedlings of *Taxus baccata* L. with a carboxylation pathway of C_3 were randomly divided into two groups. The group of seedlings that received fertilization was given 6 g of Osmocote Exact 5-6 M fertilizer (ICL, Israel) per liter in March 2014 and 2015. Conversely, the group of non-fertilized seedlings was grown without any fertilizer. The determination method of chlorophyll *a* fluorescence and the fitting of *ETR-I* response curves in *Taxus baccata* L. is the same as that of (**i**) (Robakowski et al., 2018).

(**iii**) The soybean (*Glycine max* L.) variety used is 'Zhe Xian Dou 8' with a carboxylation pathway of C_3. After germination, the soybeans are planted in pots and grown in a greenhouse with 35-40% of full sunlight. When the plant reaches a height of 40 cm, the 4th trifoliate leaf is used to measure the *ETR-I* response curve using a portable photosynthetic instrument Li-6400-40B with a fluorescent leaf chamber (Li-Cor INC. USA). During the measurement, the carbon dioxide flow rate is set at 500, the chamber temperature is maintained at 33℃, and the photosynthetically active radiation (*PAR*) is set at 2000, 1800, 1600, 1400, 1200, 1000, 700, 400, 200, 150, 100, 50 and 0 μmol photons m^{-2} s^{-1} (Ye et al., 2018).

(**iv**) The variety of *Zea mays* L. used in the experiment is 'KFJT-1' with a carboxylation pathway of C_4. After germination, the seedlings are placed in a growth chamber with a light intensity of 15000LUX and a light cycle of 13h/11h. Afer one

month of growth, one leaf is selected from each plant for the measurement of chlorophyll *a* fluorescence using a portable photosynthetic instrument Li-6800-01A with a fluorescent leaf chamber (Li-Cor INC. USA). The carbon dioxide flow rate is set at 500 μmol s^{-1}, and the relative humidity was controlled at around 70%. The gradient of light intensity was set as follows: 2000, 1800, 1600, 1400, 1200, 1000, 800, 600, 400, 200, 150, 100, 50, 25 and 0 μmol photons m^{-2} s^{-1} (Wang et al., 2022).

(**v**) The variety of *Osmanthus fragrans* Loureiro with a height of approximately 2.5 m in the campus of Jinggangshan University was chose as experimental materials. This species follows the C_3 carboxylation pathway. At noon (12:00), the surface of the leaves was exposed to a light intensity of approximately 1500 μmol m^{-2} s^{-1}, considered as sun-leaf. The surface of the leaves was exposed to a light intensity of approximately 120 μmol m^{-2} s^{-1}, considered as shaded-leaf. The chlorophyll *a* fluorescence measurements were conducted at 9:00 am using a Dual-PAM-100/F (Heinz Walz GmbH, Effeltrich, Germany). After a 30-minute dark adaptation period, a light induction of 416μmol m^{-2} s^{-1} was provided according to the instructions in the DUAl-PAM-100/F manual to measure the chlorophyll *a* fluorescence induction curve. The energy utilized in the photophysical reactions of the PSII, represented by Y(II), was directly obtained from the software provided with the instrument. The *ETR* at different intensities was calculated using the formula $ETR = PAR \times Y(\text{II}) \times 0.84 \times 0.5$ (Hu et al., 2021).

(**vi**) *Microcystis aeruginosa* FACHB905 with a carboxylation pathway of C_3 procured from the Freshwater Algae Culture Collection of the Institute of Hydrobiology, Chinese Academy of Sciences. After 8 days of cultivation using a medium with a concentration of 10 ml/g of NO_3^--N ($NaNO_3$) and 10 ml/g of NH_4^+-N (NH_4Cl), the response of *ETR-I* was measured with a Phyto-PAM fluorescence monitoring system (Walz Germany) (Yang et al., 2023).

The *ETR-I* response curves are fitted with the YRS method, and the photosynthetic parameters characterizing light-harvesting pigment molecules were calculated using the *Photosynthesis Model Simulation Software* (PMSS) (http://photosynthetic.sinaapp.com).
